# Regulation of liver receptor homologue-1 by DDB2 E3 ligase activity is critical for hepatic glucose metabolism

**DOI:** 10.1038/s41598-019-41411-x

**Published:** 2019-03-28

**Authors:** Tsai-Chun Lai, Meng-Chun Hu

**Affiliations:** Graduate Institute of Physiology, National Taiwan University College of Medicine, Taipei, 100 Taiwan

## Abstract

Liver receptor homologue-1 (LRH-1) plays a critical role in hepatic metabolism and disease. Here we show that LRH-1 protein stability is regulated by the cullin 4 (CUL4) E3 ubiquitin ligase complex. We found that DNA damage-binding protein 2 (DDB2) directly interacts with LRH-1 and functions as a substrate recognition component of CUL4-DDB1 to promote LRH-1 ubiquitination and proteasomal degradation. In human hepatoma (HepG2) cells, we observed that protein levels of endogenous LRH-1 are increased by insulin without a change in mRNA levels of *LRH*-1. However, overexpression of DDB2 impaired the insulin-stimulated increase in LRH-1 levels. In addition, DDB2 overexpression decreased LRH-1 transcriptional activation and expression of target genes, such as glucokinase, whereas knockdown of DDB2 increased the expression of glucokinase. Finally, we demonstrated that DDB2 knockdown increases glucose uptake and intracellular levels of glucose-6-phosphate in HepG2 cells. Our study reveals a novel regulatory mechanism of LRH-1 activity and suggests a role for DDB2 in hepatic glucose metabolism.

## Introduction

Liver receptor homologue-1 (LRH-1; NR5A2) is an orphan member of the nuclear receptor NR5A subfamily. LRH-1 is predominantly expressed in enterohepatic tissues and the ovary, and drives the expression of genes involved in development, metabolism, steroidogenesis, and proliferation^1^. LRH-1 is highly expressed in the liver and regulates a variety of metabolic pathways, including bile acid synthesis, reverse cholesterol transport, and fatty acid synthesis^2–5^. LRH-1 is involved in the control of hepatic glucose and lipid metabolism by regulating the expression of glucokinase (GCK)^6^. Consequently, glucose-induced glycolysis, glycogen synthesis, and fatty acid synthesis are reduced in liver-specific LRH-1 knockout mice^6^. In addition, LRH-1 has been shown to induce an UPR (unfolded protein response)-independent pathway that mediates the response to liver endoplasmic reticulum stress resolution^7^.

Transcription factors regulate gene expression in response to environmental change, therefore their activity is under a fine control to regulate the transcription of specific genes. Similar to most nuclear receptors, LRH-1 can interact with co-regulators, such as multiprotein bridging factor 1, steroid receptor coactivator 1, and short heterodimer partner, which either enhances or represses its transcriptional activity^8–10^. Moreover, the activity of LRH-1 is modulated by post-translational modification. Phosphorylation of the hinge domain by 4β-phorbol 12-myristate 13-acetate (PMA) stimulates the transactivation of LRH-1^11^. In addition, LRH-1 is conjugated to small ubiquitin-related modifier (SUMO), which leads to the repression of its transcriptional activity and localization of LRH-1 in inactive nuclear bodies^11–13^. Protein turnover through ubiquitin-proteasome system (UPS) is also a prominent mechanism that controls the levels of transcription factors^14^. Several nuclear receptors are ubiquitinated and the UPS-mediated proteolysis is involved in the regulation of nuclear receptor-mediated gene transcription^15^. For instance, ligand binding is known to activate transactivation and induce proteasome-dependent proteolysis of several nuclear receptors, including estrogen receptor and progesterone receptor^16,17^. Moreover, certain ubiquitin proteasome components, such as E6-AP and MDM2, have been identified as coactivators for nuclear hormone receptors^18,19^. The ubiquitin-proteasome pathway is believed to regulate the nuclear receptor-mediated transcription; however, the molecular mechanisms that control LRH-1 levels remain unclear.

Proteins that are targeted for degradation by the 26S proteasome are covalently tagged with ubiquitin. The conjugation of ubiquitin involves a series of reactions of three classes of enzymes; ubiquitin-activating enzyme (E1), ubiquitin-conjugating enzymes (E2), and ubiquitin-protein ligase (E3)^20^. The E3s confer the selection and specificity for protein ubiquitination and thus large amounts of E3s exist in an organism. The cullin-RING ubiquitin ligases (CRLs) are the largest subclass of E3s and control the ubiquitination of a wide variety of substrates. Human cells contain seven cullin family members (CUL1, 2, 3, 4A, 4B, 5, and 7), each of which serve as a scaffold for the assembly of many distinct multi-subunit CRL complexes^21^. The C-terminus of cullins interacts with a small RING finger protein, ROC1 (regulator of cullins 1) or ROC2, to recruit E2 enzyme and to assemble distinct substrate recruiting modules at their N-terminus^22^. These modules typically consist of an adaptor protein and a variable receptor protein that are responsible for substrate recognition.

CUL4A and CUL4B are two closely related paralogs in mammalian cells. The CRL4 complex is composed of the scaffold CUL4A or CUL4B, RING protein ROC1 and the adaptor DNA damage binding-protein 1 (DDB1)^23^. Numerous proteins containing WD40 domain are CUL4-DDB1 associated factors (DCAFs) that facilitate the binding of substrates to CUL4-DDB1 complex for ubiquitination^24^. CRL4 substrates involve the DNA replication licensing factor CDT1^25^, repair factor XPC^26^, histones H2A/H3/H4^27,28^ and cyclin E^29^, indicating an important role of CUL4 in the maintenance of genomic stability and cell cycle regulation. CUL4A overexpression has been reported in several human cancers^30–32^, while CUL4B mutations were identified in patients with X-linked mental retardation (XLMR)^33^. CUL4-RING ligase has been shown to regulate the stability of nuclear receptors, such as estrogen receptor (ER) and androgen receptor (AR)^34,35^. In this study, we identified DNA damage-binding protein 2 (DDB2) as a novel regulator of LRH-1. DDB2 interacts with LRH-1 and promotes its degradation through the CUL4-DDB1 complex. Our results show that DDB2 inhibits LRH-1-mediated gene expression and plays a critical role in hepatic glucose regulation.

## Results

### LRH-1 is degraded by the ubiquitin-proteasome system

**Figure 1:**
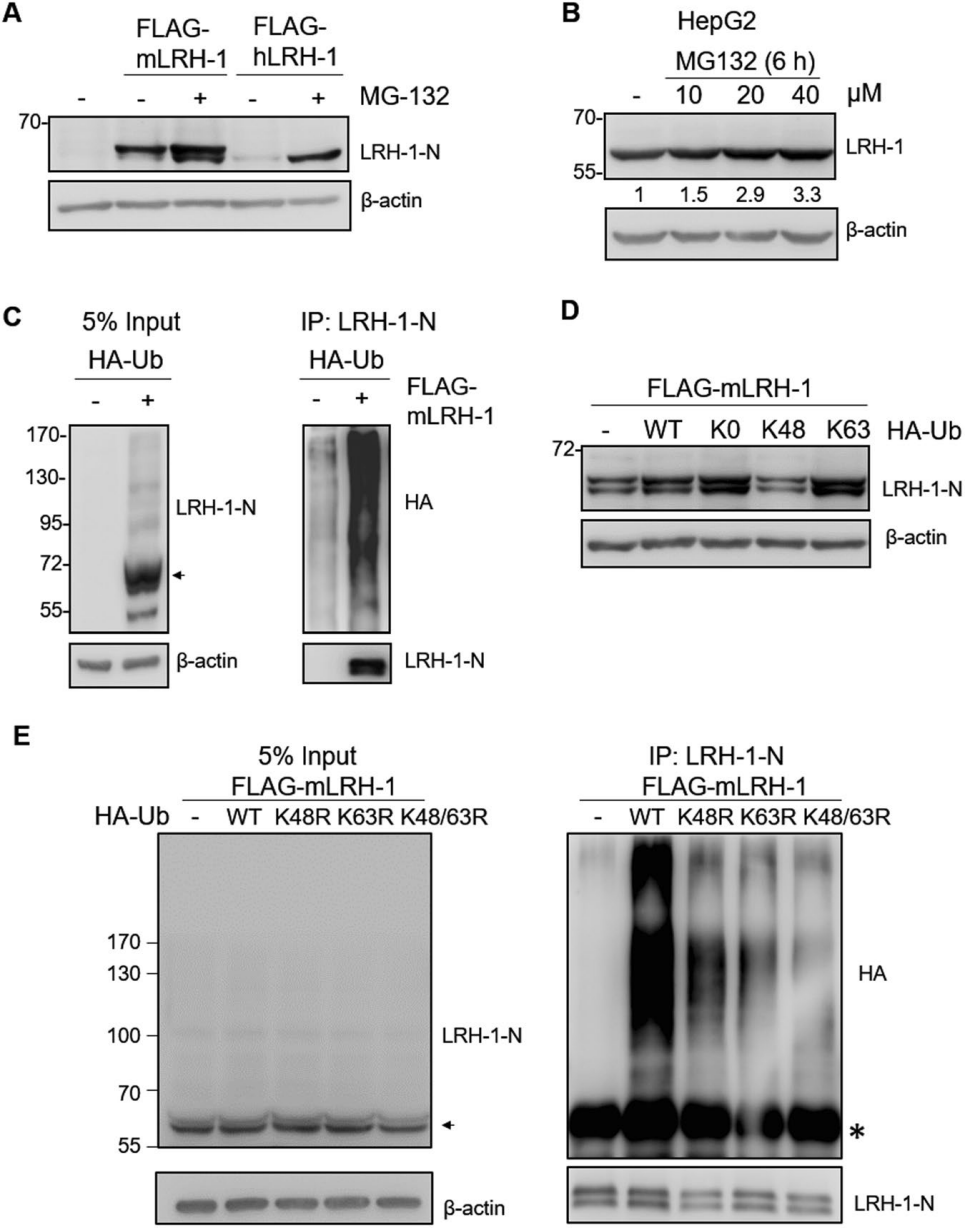
LRH-1 protein is degraded by the ubiquitin proteasome pathway. (**A**) FLAG-mLRH-1 or FLAG-hLRH-1 was transfected into HEK293T cells. After 24 h, cells were treated with DMSO (vehicle control) or MG-132 (10 μM) for 24 h. Cell lysates were analyzed by immunoblotting with the anti-LRH-1-N antibody. (**B**) HepG2 cells were serum-starved overnight and treated with MG-132 (0–40 μM) for 6 h. Protein levels of endogenous LRH-1 were detected by immunoblotting with the anti-LRH-1 antibody. Numbers below the blot are the relative densitometric values normalized to those of β-actin. (**C**) FLAG-mLRH-1 or empty vector (−) was co-transfected with HA-Ub into HEK293T cells. After 24 h, cells were treated with MG-132 (10 μM) for 24 h. Cell lysates were immunoprecipitated with the anti-LRH-1-N antibody and immunoblotted with the anti-LRH-1-N or anti-HA. (**D**) FLAG-mLRH-1 was co-transfected with empty vector (−) or HA-Ub [wild type (WT), lysine-less Ub mutant (K0), lysine 48 Ub mutant (K48), or lysine 63 Ub mutant (K63)] into HEK293T cells. Cell lysates were analyzed by immunoblotting with the anti-LRH-1-N antibody. (**E**) FLAG-mLRH-1 was co-transfected with HA-Ub [WT, lysine 48 to arginine Ub mutant (K48R), lysine 63 to arginine Ub mutant (K63R), or double lysines mutant (K48/63R)] or empty vector in HEK293T cells. After 24 h, cells were treated with MG-132 (10 μM) for 24 h. Cell lysates were immunoprecipitated with the anti-LRH-1-N antibody and immunoblotted with the anti-LRH-1-N or anti-HA antibodies. Asterisk indicates heavy chain IgG background. Uncropped images of blots were shown in Supplementary Fig. S5.

To determine whether LRH-1 is degraded via the proteasome pathway, we examined the effect of proteasome inhibition on LRH-1 levels. Inhibition of proteasome activity by MG132 increased the protein levels of exogenous mouse and human LRH-1 in HEK293T cells (Fig. 1A). We further used the HepG2 (human hepatoma) cell line, which endogenously expressed LRH-1, to analyze the influence of MG132 on LRH-1. The results showed that MG132 treatment resulted in a concentration-dependent increase in levels of endogenous LRH-1 protein in HepG2 cells (Fig. 1B). Targeting of proteins for proteasomal degradation is mainly mediated by ubiquitination. To verify whether LRH-1 protein was ubiquitinated, HEK293T cells were co-transfected with mLRH-1 and HA-tagged ubiquitin (HA-Ub) constructs. After immunoprecipitation against LRH-1, the high-molecular-weight smear of ubiquitinated proteins were detected by HA immunoblotting (Fig. 1C), revealing that LRH-1 was polyubiquitinated in HEK293T cells. The Lys48-linked polyubiquitin chain is the principal signal for targeting of proteins to proteasomal degradation. Expression of wild-type (WT), and mutant ubiquitin variants like lysine-less (Ub-K0), lysine 48 conjugated (Ub-K48), and lysine 63 conjugated (Ub-K63) ubiquitin in HEK293T cells, revealed that Ub-K0 and Ub-K63 caused an increase in LRH-1 levels, whereas Ub-K48 appreciably reduced the amount of LRH-1 protein (Fig. 1D), suggesting that K48 conjugation promoted LRH-1 protein degradation. Co-transfection of HEK293T cells with the single K48R mutant ubiquitin construct and the LRH-1 construct, disrupted the Lys48-linked polyubiquitin chain, reducing ubiquitination levels of mLRH-1 as compared to those caused by wild-type ubiquitin (Fig. 1E). Similarly, co-transfection of HEK293T cells with the single K63R mutant construct and the LRH-1 construct also led to a decrease in mLRH-1 ubiquitinated protein, and this reduction was more pronounced in the double K48/63R mutant of ubiquitin. These data implicated that LRH-1 is ubiquitinated via Lys48 and Lys63 linkages and that Lys48-linked ubiquitin enhances LRH-1 protein degradation by proteasome. LRH-1 also can be targeted for sumoylation on lysine residues^12,13^. To test the potential association between ubiquitnation and sumoylation, the major sumoylation site K289 and several potential target lysine residues, K173, K213, K329, and K389, were mutated to arginine, and the mutants were transfected into HEK293T cells. As observed in wild type, MG132 treatment efficiently increased the protein levels in the mutants (Supplemental Fig. S1), suggesting that these lysine residues are not critical for LRH-1 ubiquitination.

### Cullin 4-RING ubiquitin ligases regulate LRH-1 proteolysis

**Figure 2:**
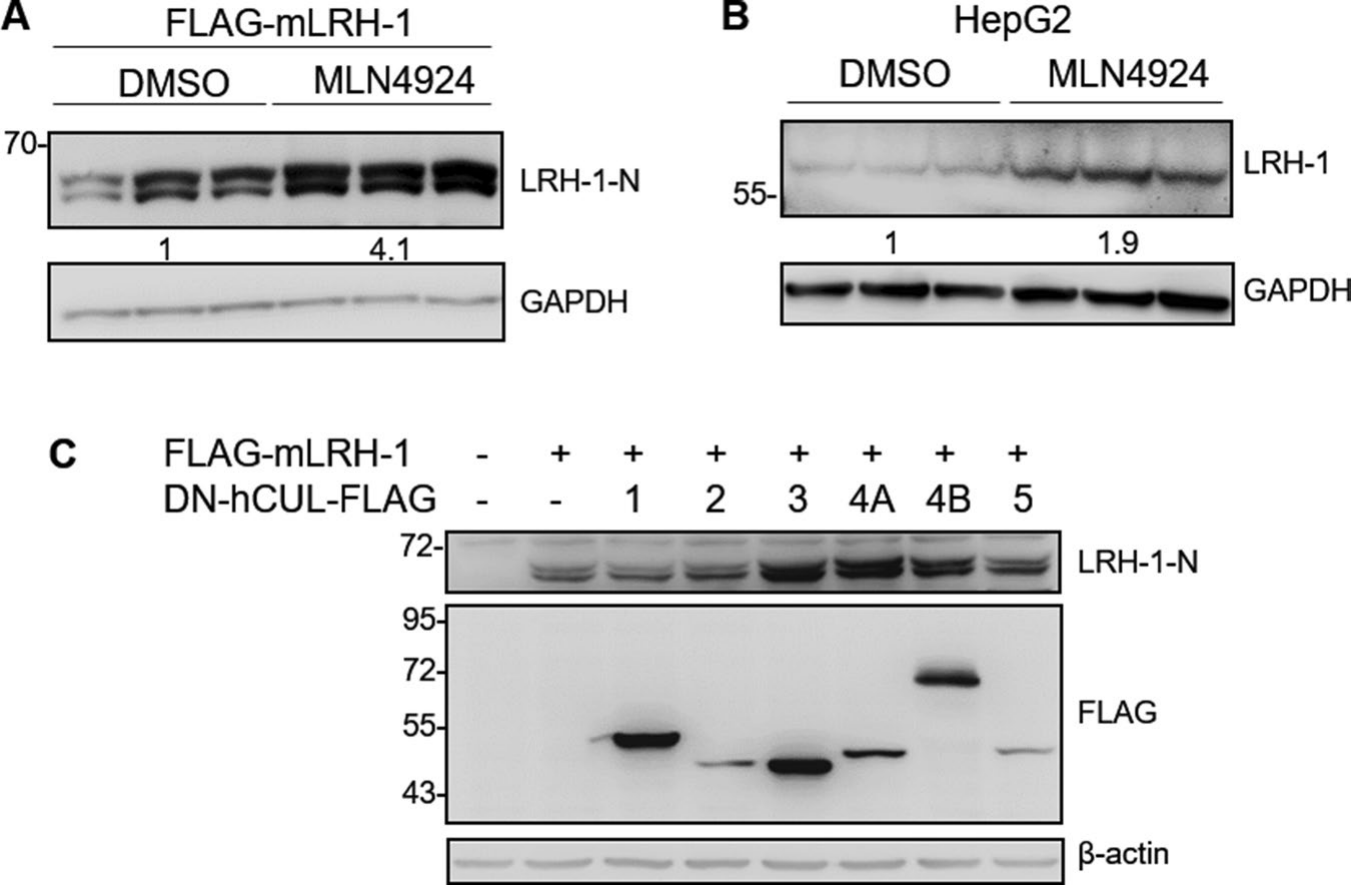
CRL4 ubiquitin ligases regulate LRH-1 protein degradation. (**A**) FLAG-mLRH-1 was transfected into HEK293T cells. After 24 h, cells were treated with DMSO (vehicle control) or MLN4924 (10 μM) for 24 h. Cell lysates were analyzed by immunoblotting with the anti-LRH-1-N antibody. Numbers below the blot are the average of densitometric values normalized to those of GAPDH. (**B**) HepG2 cells were serum-starved overnight and treated with DMSO or MLN4924 for 24 h. Protein levels of endogenous LRH-1 were determined by immunoblotting with the anti-LRH-1 antibody. Numbers below the blot are the average of densitometric values normalized to those of GAPDH. (**C**) FLAG-mLRH-1 was co-transfected with FLAG-tagged dominant negative constructs of Cullin 1, 2, 3, 4A, 4B, and 5 or empty vector (−) into HEK293T cells. After 48 h, cell lysates were subjected to immunoblotting analysis with anti-LRH-1-N or anti-FLAG antibodies. β-actin was used as the loading control. Uncropped images of blots were shown in Supplementary Fig. S6.

Given that cullin-RING ligases (CRLs) are the largest family of E3 ubiquitin ligases, we investigated whether the degradation of LRH-1 is regulated by CRLs. MLN4924 is a specific inhibitor of NEDD8-activating enzyme that blocks cullin neddylation and leads to a loss of the cullin ubiquitin ligase activity. HEK293T cells were transfected with LRH-1 and then treated with a vehicle (DMSO) or MLN4924 for 24 h. We found that inhibition of CRL activity by MLN4924 increased the protein levels of mLRH-1 (Fig. 2A) and hLRH-1 (Supplementary Fig. S2A) in HEK293T cells. Similarly, treatment with MLN4924 increased the protein levels of endogenous LRH-1 by approximately 1.9-fold in HepG2 cells (Fig. 2B), indicating that CRLs are involved in the regulation of LRH-1 proteolysis.

To identify the specific CRL responsible for the degradation of LRH-1, HEK293T cells were co-transfected with FLAG-LRH-1 and various dominant-negative (DN) cullin constructs, which contain truncated C-terminus impairing the interaction with E2^36^. The immunoblotting results showed that protein levels of both mouse and human LRH-1 were increased in cells co-transfected with DN-CUL4A or DN-CUL4B (Fig. 2C and Supplementary Fig. S2B). Co-transfection of the DN-CUL3 also increased the protein levels of mLRH-1, but not hLRH-1. These data suggested that LRH-1 levels were regulated by CRL4.

### CUL4-DDB1-DDB2 ubiquitin ligase complexes govern LRH-1 protein levels

**Figure 3:**
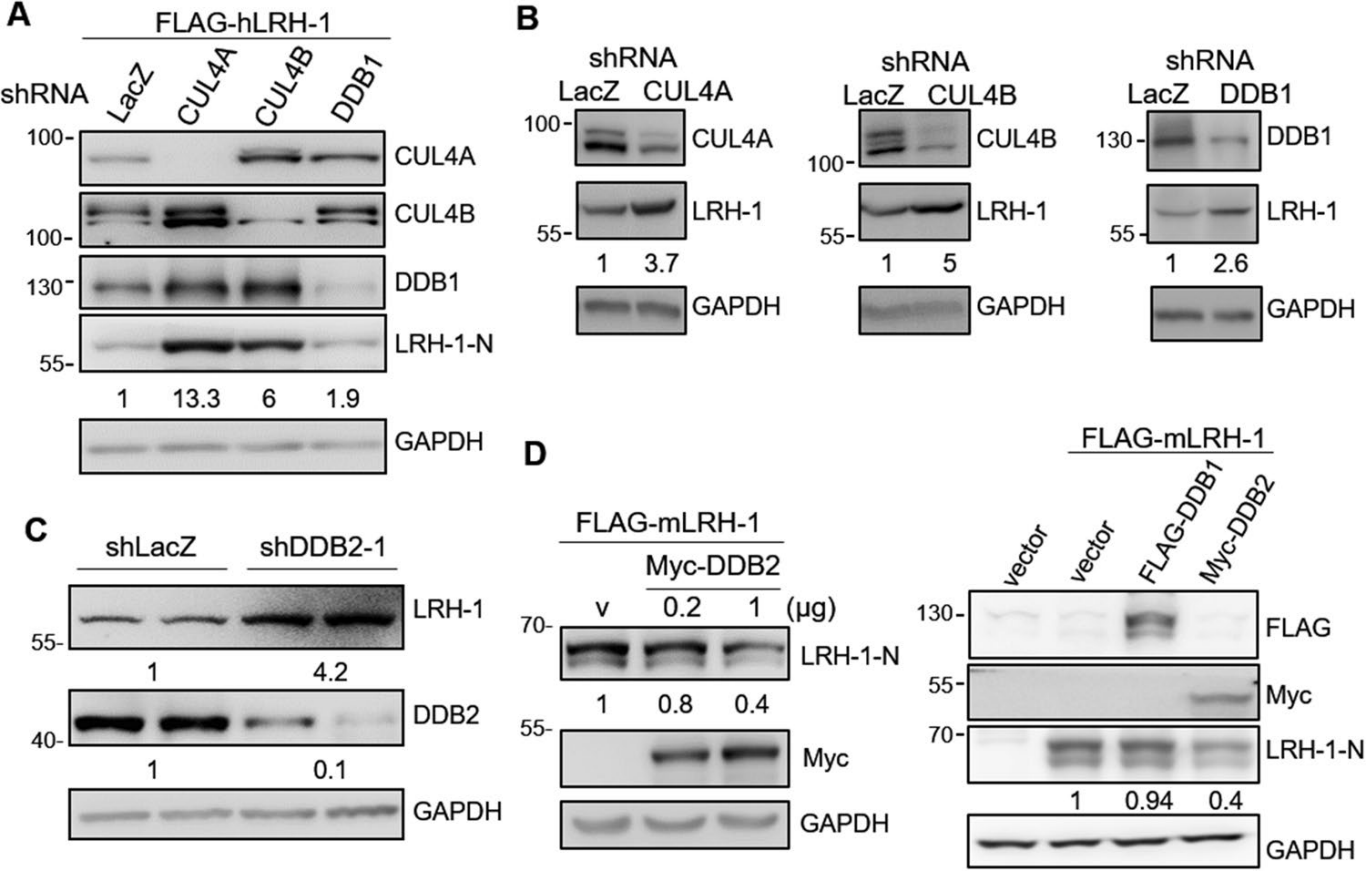
CUL4-DDB1-DDB2 ubiquitin ligase complex affects LRH-1 protein degradation. (**A**) HEK293T cells transduced with shRNA specific for CUL4A, CUL4B, DDB1 or LacZ (negative control) were transfected with FLAG-hLRH-1. Cell lysates were analyzed by immunoblotting with antibodies as specified. (**B**) HepG2 cells were transduced with the specified shRNA and cell lysates were analyzed by immunoblotting with antibodies as specified. (**C**) HepG2 cells were transduced with shRNA specific for DDB2 or LacZ. Endogenous protein levels were analyzed by immunoblotting with anti-LRH-1 or anti-DDB2 antibodies. (**D**) FLAG-mLRH-1 was co-transfected with Myc-DDB2, FLAG-DDB1 or vector into HEK293T cells. After 48 h, cell lysates were subjected to immunoblotting analysis with anti-LRH-1-N, anti-Myc or anti-FLAG antibodies. Numbers below the blot are the relative densitometric values normalized to those of GAPDH. Uncropped images of blots were shown in Supplementary Fig. S7.

CRL4 complexes contain core component CUL4A or CUL4B, RING finger protein ROC1, and the adaptor protein DDB1. To further assess the regulatory role of CRL4 on LRH-1 levels, we performed knockdown experiments using lentiviral shRNA for CUL4A, CUL4B, DDB1, or control (LacZ) in HEK293T cells. As seen in Fig. 3A, the levels of transfected FLAG-hLRH-1 increased when CUL4A, CUL4B, or DDB1 were knocked down in HEK293T cells. Furthermore, knockdown of CUL4A, CUL4B or DDB1 expression in HepG2 cells also increased the endogenous LRH-1 protein levels (Fig. 3B).

CUL4-DDB1 interacts with a family of WD40-repeat proteins that is responsible for substrate recognition^24^. One of the best-characterized substrate receptors is DDB2, which has been shown to mediate the degradation of androgen receptor, a member of nuclear receptors^34^. Hence, we examined whether DDB2 is a potential substrate receptor involved in LRH-1 degradation. We found that knockdown of DDB2 increased endogenous LRH-1 protein levels in HepG2 cells (Fig. 3C). In contrast, co-expression with Myc-DDB2 decreased the protein levels of mLRH-1 in a concentration-dependent manner in HEK293T cells, whereas co-expression with FLAG-DDB1 had no detectable effect on LRH-1 protein levels (Fig. 3D). These results suggest that DDB2 is a specific substrate receptor for LRH-1.

### LRH-1 interacts with DDB2

**Figure 4:**
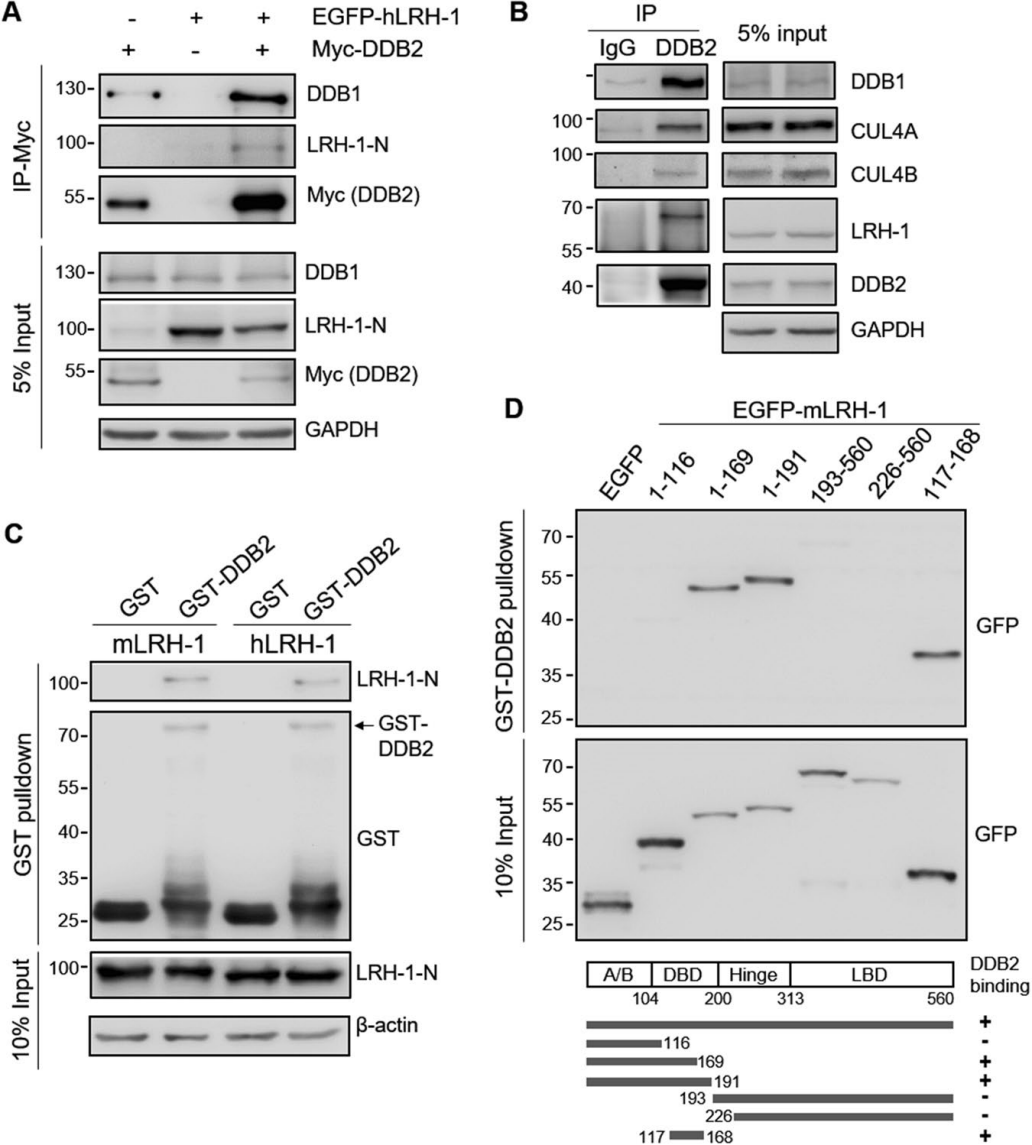
LRH-1 interacts with DDB2 through its DNA binding domain. (**A**) EGFP-hLRH-1 and/or Myc-DDB2 or empty vector were transfected into HEK293T cells. After 42 h, cells were incubated with MG-132 (10 μM) for 6 h, and cell lysates were immunoprecipitated with the anti-Myc antibody and immunoblotted with antibodies as indicated. (**B**) HepG2 cell lysates were immunoprecipitated with the anti-DDB2 antibody or control (IgG) antibody and immunoblotted with antibodies as indicated. (**C**) EGFP-mLRH-1 or EGFP-hLRH-1 were transfected into HEK293T cells. After 48 h, cell lysates were incubated with bead-bound GST or GST-DDB2. Bound protein was subjected to immunoblotting with anti-LRH-1-N or anti-GST antibodies. (**D**) EGFP-mLRH-1 truncated constructs or empty vectors were transfected into HEK293T cells. After 48 h, cell lysates were incubated with bead-bound GST-DDB2 and immunoblotted with the anti-GFP antibody. The results are summarized below the blot. Uncropped images of blots were shown in Supplementary Fig. S8.

To test whether LRH-1 interacts with DDB2, EGFP-hLRH-1 and Myc-DDB2 were transfected alone or together into HEK293T cells. Cells were treated with MG132 for 6 h and cell lysates were immunoprecipitated with an anti-Myc antibody, followed by immunoblot analysis. As shown in Fig. 4A, EGFP-hLRH-1 was co-precipitated with Myc-DDB2, indicating that hLRH-1 could interact with DDB2. We also observed that endogenous DDB1 was co-precipitated with Myc-DDB2. However, an interaction between EGFP-hLRH-1 and DDB1, CUL4A, or CUL4B was not detected by the co-immunoprecipitation assay in transfected HEK293T cells (data not shown). To further determine whether endogenous LRH-1 and DDB2 could interact, HepG2 cell lysates were immunoprecipitated with anti-DDB2 antibody. Immunoblot analysis revealed that endogenous LRH-1 co-precipitated with DDB2 (Fig. 4B). As expected, CUL4A, CUL4B and DDB1 were also detected in the immunoprecipitate, indicating that DDB2 is associated with CUL4-DDB1 complex. We further performed GST pull-down assays to examine the interaction between LRH-1 and DDB2 *in vitro*. Both mouse and human LRH-1 were pulled down by GST-DDB2, but not by the GST control (Fig. 4C), indicating the specificity of interaction. To map the domains of LRH-1 involved in the interaction with DDB2, we developed a series of mLRH-1 truncated constructs. The results showed that EGFP-LRH-1_1–169_, EGFP-LRH-1_1–191_ and EGFP-LRH-1_117–168_ associated with GST-DDB2, whereas EGFP-LRH-1_1–116_, EGFP-LRH-1_193–560_ and EGFP-LRH-1_226–520_ exhibited no interaction with GST-DDB2 in the pull-down assay (Fig. 4D). This indicates that LRH-1 directly interacts with DDB2 through the DNA binding domain (DBD) amino acid 117–168. Together, these data suggest that DDB2 serves as a substrate receptor for recognizing LRH-1 and targets LRH-1 to the CUL4-DDB1 complex for degradation.

We then examined the cellular localization of LRH-1 and DDB2 by immunostaining. As seen in Supplementary Fig. S3, LRH-1 was exclusively localized in the nucleus, while DDB2 was present in both the nucleus and cytoplasm. Merged images showed significant co-localization of LRH-1 and DDB2 in the nucleus.

### DDB2 facilitates LRH-1 ubiquitination and destabilizes LRH-1

**Figure 5:**
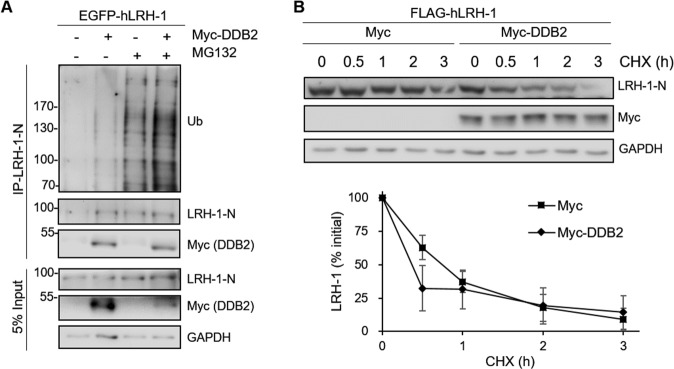
DDB2 promotes LRH-1 ubiquitination and destabilizes LRH-1 protein turnover. (**A**) EGFP-hLRH-1 was co-transfected with empty vector (−) or Myc-DDB2 into HEK293T cells. After 42 h, cells were treated with DMSO or MG-132 (10 μM) for 6 h. Cell lysates were immunoprecipitated with the anti-LRH-1-N antibody and immunoblotted with indicated antibodies. (**B**) FLAG-hLRH-1 was co-transfected with empty vector or Myc-DDB2 into HEK293T cells. After 24 h, cells were treated with 100 μg/ml cycloheximide (CHX) for the specified periods of time. Cell lysates were immunoblotted with anti-LRH-1-N or anti-Myc antibodies. Quantification of the levels of LRH-1 protein was done by normalizing densitometric values to those of GAPDH. Values were mean ± SEM of four independent experiments. Uncropped images of blots were shown in Supplementary Fig. S9.

To determine whether DDB2 affects the ubiquitination status of LRH-1, EGFP-hLRH-1 together with control vector or Myc-DDB2 were transfected into HEK293T cells and cell lysates were immunoprecipitated with an anti-LRH-1-N antibody. Immunoblot analysis showed that MG132 treatment promoted the accumulation of ubiquitinated LRH-1 and DDB2 apparently increased the ubiquitination level of LRH-1 as compared to that of the empty vector control (Fig. 5A). We next investigated the regulation of LRH-1 protein turnover by DDB2 by using cycloheximide (CHX)-based pulse-chase assays. As shown in Fig. 5B, the protein stability of hLRH-1 is markedly reduced in the presence of DDB2. These data suggest that DDB2 promotes the ubiquitin-dependent degradation of LRH-1.

### Insulin upregulates LRH-1 repressed by DDB2 in HepG2 cells

**Figure 6:**
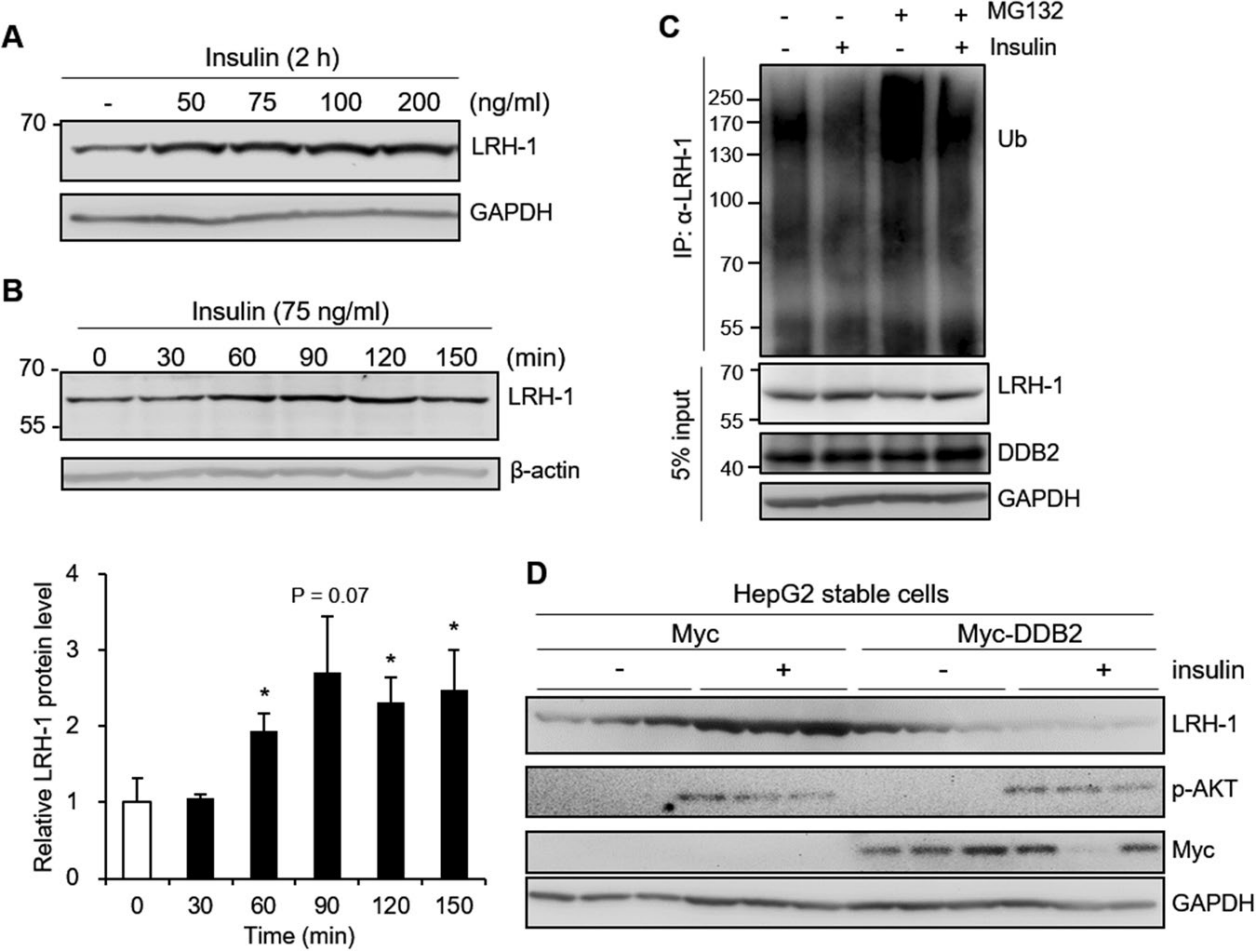
Insulin increases LRH-1 protein levels. (**A**) HepG2 cells were serum-starved overnight, and treated with various concentrations of insulin for 2 h. Cell lysates were immunoblotted with the anti-LRH-1 antibody. (**B**) HepG2 cells were serum-starved overnight and incubated with 75 ng/ml insulin for specified periods of time. Protein levels of endogenous LRH-1 were determined by immunoblotting with the anti-LRH-1 antibody and quantified by normalizing densitometric values to those of β-actin. Data are expressed as mean ± SEM relative to control 0 h of three independent experiments (lower panel). *P < 0.05 compared to control. (**C**) HepG2 cells were serum-starved overnight, followed by MG-132 (10 μM) treatment for 30 minutes prior to insulin (100 nM) incubation for 2 h. Cell lysates were immunoprecipitated with the anti-LRH-1 antibody and immunoblotted with indicated antibodies. (**D**) HepG2 cells stable for Myc-DDB2 or control Myc were serum-starved for overnight and treated with 200 ng/ml insulin for 90 minutes. Cell lysates were analyzed by immunoblotting with antibodies as indicated. Uncropped images of blots were shown in Supplementary Fig. S10.

LRH-1 regulates the expression of glucokinase (GCK) gene, which controls the key step for glycolysis and glycogen synthesis, and thus is involved in hepatic glucose metabolism^6^. Since insulin is the primary positive regulator for liver GCK gene expression^37^, we investigated the potential role of insulin in regulation of LRH-1 expression. HepG2 cells were incubated with insulin (50~200 ng/ml) for 2 h and results showed that LRH-1 protein levels increased by about 2-fold after insulin treatment (Fig. 6A). Sixty minutes after treatment, the induction of LRH-1 protein levels by insulin was observed in HepG2 cells (Fig. 6B). To determine the effects of insulin on LRH-1 mRNA expression, HepG2 cells were incubated with insulin (75 ng/ml) for various time periods (1~6 h). The quantitative RT-PCR analysis revealed that no significant change in LRH-1 mRNA levels was found after insulin treatment (Supplementary Fig. S4). To further explore whether insulin influences LRH-1 protein stability, we analyzed the ubiquitination of LRH-1. HepG2 cells were treated with or without insulin for 2 h in the presence or absence of MG132 pre-incubation. Endogenous LRH-1 was immunoprecipitated using anti-LRH-1 antibody and ubiquitinated LRH-1 was detected with an anti-ubiquitin antibody by immunoblot analysis. The results showed that insulin treatment decreased poly-ubiquitination of LRH-1 both in the presence or absence of MG132 (Fig. 6C). These results suggested that reduced ubiquitination and degradation of LRH-1 by insulin is a likely cause for the increase of LRH-1 protein.

Our results demonstrated that DDB2 mediates the degradation of LRH-1. To determine whether DDB2 affects insulin regulation of LRH-1 protein expression, we stably overexpressed Myc-DDB2 protein in the HepG2 cell line. Overexpression of DDB2 in stable cells was confirmed by immunoblotting with anti-Myc antibody (Fig. 6D). As shown in Fig. 6D, the ability of insulin to stimulate LRH-1 protein levels was suppressed in HepG2 cells stably expressing DDB2. Thus, DDB2 may act as a negative regulator affecting the normal function of LRH-1 in HepG2 cells.

### Effects of DDB2 on LRH-1-mediated functions

**Figure 7:**
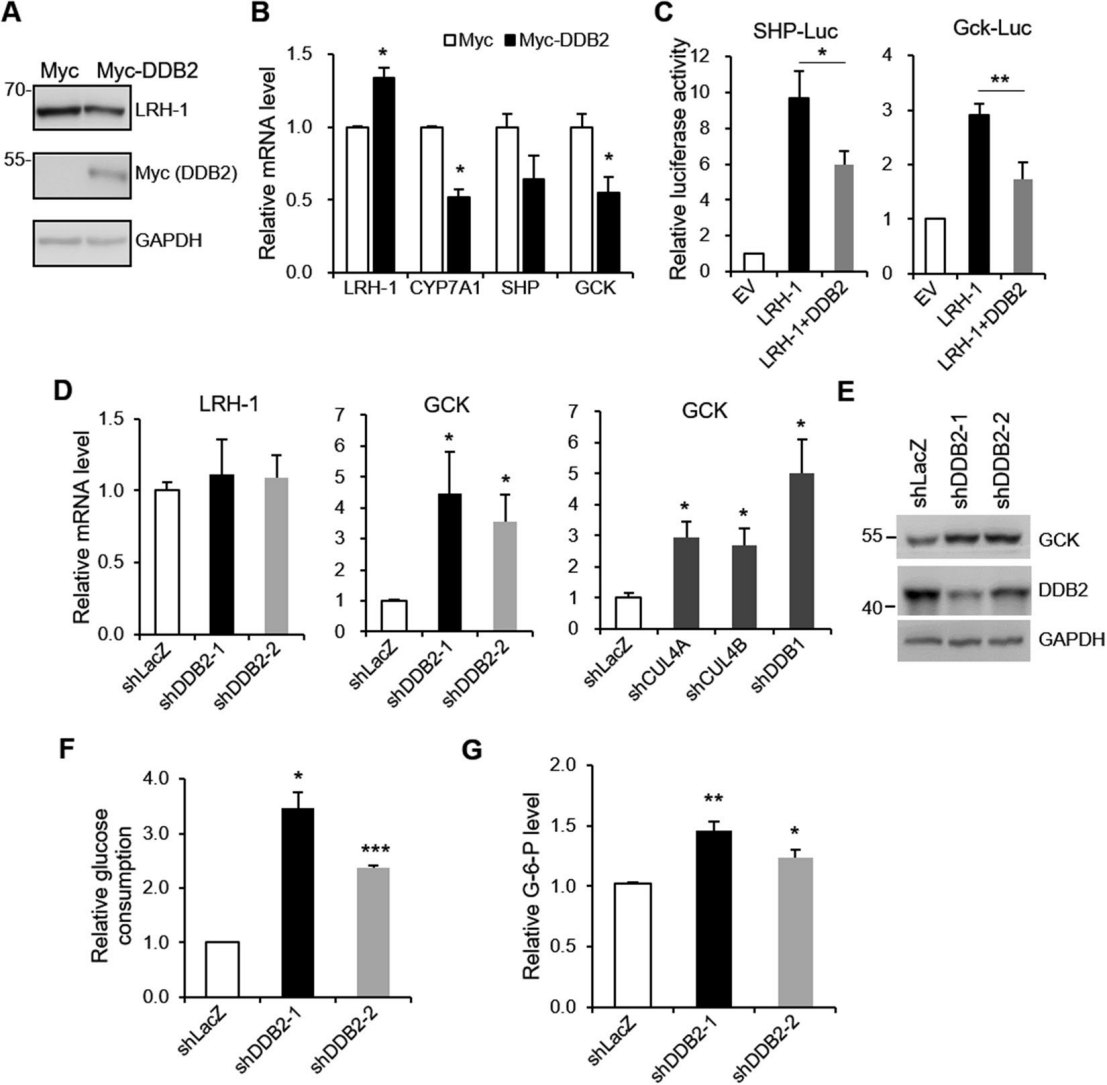
Effects of DDB2 on LRH-1-mediated gene expression and glucose metabolism. (**A**) Myc-DDB2 or empty control vector (Myc) was transfected into HepG2 cells. Protein levels of endogenous LRH-1 and Myc-DDB2 were analyzed by immunoblotting. (**B**) mRNA levels of LRH-1, CYP7A1, SHP, and GCK were quantified by RT-qPCR. Data are expressed as mean ± SEM relative to control of four independent experiments. *P < 0.05 compared to control. (**C**) The SHP or Gck promoter-luciferase construct was co-transfected with plasmids expressing indicated proteins or control empty vector (EV) into HEK293T cells. After 24 h, luciferase activity was measured. Data are represented as mean ± SEM of five independent experiments. *P < 0.05. (**D**~**F**) HepG2 cells were transduced with shRNA specific for DDB1, DDB2, CUL4A, CUL4B or control LacZ as indicated. (**D**) mRNA levels of LRH-1 and GCK were quantified by RT-qPCR (n = 6). (**E**) Protein levels were determined by immunoblotting with the indicated antibody. (**F**) Glucose consumption was analyzed (n = 3). (**G**) Glucose-6-phosphate levels were measured (n = 5). Data are expressed as mean ± SEM. *P < 0.05, **P < 0.001, ***P < 0.0001 compared to control. Uncropped images of blots were shown in Supplementary Fig. S11.

As DDB2 decreases LRH-1 protein stability, we evaluated the effect of DDB2 on LRH-1 target gene transcription. Overexpression of DDB2 reduced the protein levels of LRH-1 in HepG2 cells (Fig. 7A). However, the mRNA levels of LRH-1 were slightly increased in DDB2-overexpressed HepG2 cells (Fig. 7B). This effect may represent a compensatory response to reduced LRH-1 protein levels. Overexpression of DDB2 showed a decreased expression of LRH-1 target genes, CYP7A1, small heterodimer partner (SHP), and glucokinase (GCK) (Fig. 7B). We further examined the effect of DDB2 on LRH-1-mediated activation of the SHP and Gck promoters. As shown in Fig. 7C, co-expression of DDB2 decreased the LRH-1-stimulated SHP and Gck promoter activity. In contrast, knockdown of DDB2 in HepG2 cells resulted in an increase in GCK at both the mRNA and protein levels but had no effect on LRH-1 expression (Fig. 7D,E). Additionally, knockdown of CUL4A, CUL4B, or DDB1 increased the GCK mRNA levels in HepG2 cells (Fig. 7D). These data suggest that DDB2 can regulate LRH-1-mediated gene transcription.

Next, we analyzed the effects of DDB2 on glucose metabolism. As shown in Fig. 7F, knockdown of DDB2 expression increased glucose consumption in HepG2 cells. Hepatic glucokinase facilitates the conversion of glucose into glucose-6-phosphate, which plays a central role in glucose homeostasis. Consistent with the increased levels of glucose uptake, we observed that HepG2 cells with DDB2 knockdown display elevated intracellular levels of glucose-6-phosphate, which is formed by the phosphorylation of glucose (Fig. 7G). The results suggested that DDB2 may have physiological significance in regulation of glycolysis.

## Discussion

In the present study, we first demonstrated that LRH-1 is polyubiquitinated and targeted for degradation by the proteasome. We found that the CUL4-based complex functions as the E3 ubiquitin ligase involved in the regulation of LRH-1 degradation. DDB2 is a WD40 protein and exists as a heterodimeric complex with DDB1, which is involved in DNA damage recognition in the nucleotide excision repair (NER) pathway^38,39^. DDB2 is known to function as the substrate receptor of CUL4-DDB1 ligase to promote the ubiquitination of histones H2A, H3, H4 and of the NER protein XPC at sites of UV-induced DNA damage^26–28^. DDB2 itself is also a target of CUL4-DDB1 ligase for ubiquitination and degradation^40^. Here, we demonstrated that DDB2 directly interacts with LRH-1 (Fig. 4) and facilitates LRH-1 protein ubiquitination and degradation (Fig. 5). Overexpression of DDB2 decreased LRH-1 protein levels, whereas knockdown of DDB2 notably increased LRH-1 protein levels (Fig. 3). Therefore, DDB2 may act as the substrate receptor to mediate LRH-1 degradation via the CUL4-DDB1 ligase complex and has an impact on LRH-1-mediated metabolic activity.

Growing evidence has shown that E3 ubiquitin ligases play a vital role in glucose homeostasis. For example, several E3 ubiquitin ligases, such as mitsugumin 53 (MG53), Casitas B-lineage lymphoma b (Cblb), CRL7, and F-box only protein 2 (FBXO2), promote insulin receptor (IR) or insulin receptor substance (IRS)-1/2 for ubiquitin-dependent degradation and therefore impair insulin signaling^41–44^. The transcription factor Forkhead Box O1 (FOXO1) is targeted for proteasomal degradation by constitutive photomorphogenic 1 (COP1) that leads to the inhibition of FOXO1-mediated gluconeogenesis by insulin^45^. It has been recently reported that the CUL4-DDB1 complex plays a role in glucose homeostasis, where CUL4A-DDB1 enhances FOXO1 stability by inducing the degradation of circadian protein cryptochrome 1 (CRY1) and thereby promotes FOXO1-mediated gluconeogenesis^46^. A previous study has shown that the CUL4A-DDB1 targets the degradation of CRY1 via the substrate receptor Cdt10-dependent transcript 2 (CDT2)^47^. Moreover, CUL4B-DDB1 was found to promote the degradation of peroxisome proliferator-activated receptor γ (PPARγ) and thus CUL4B deletion in adipocytes led to enhanced adipogenesis^48^. LRH-1 functions as a transcriptional activator of GCK to regulate glucose and lipid metabolism in the liver^6^. We have demonstrated that DDB2 significantly inhibited LRH-1-stimulated gene expression including GCK and DDB2 knockdown increased glucose uptake in HepG2 cells. In addition, insulin-stimulated LRH-1 protein levels were decreased by co-expression with DDB2, implying that DDB2 is involved in the regulation of hepatic glucose metabolism, at least, partially, via mediation of LRH-1 protein stability. Our results suggest a previously unidentified role for DDB2 in the regulation of hepatic glucose metabolism. Collectively, these findings indicate the important role of CRL4 in modulation of metabolic homeostasis by targeting multiple regulatory proteins for degradation by recruiting different substrate recognition components, including DDB2.

LRH-1 plays essential roles in several metabolic processes in the liver; however, little is known about the regulation of LRH-1 expression under physiological conditions. In this study, we found that insulin elevated LRH-1 protein levels in a time- and dose-dependent manner in HepG2 cells (Fig. 6). This was not due to the upregulation of LRH-1 mRNA expression (Supplementary Fig. S4). Insulin can rapidly stimulate protein synthesis by activation of several translational initiation and elongation factors through phosphoinositide 3-kinase^49^. In addition, insulin exhibits a significant effect on inhibition of protein degradation, primarily through suppression of the ubiquitin-proteasome pathway^50^. Insulin deficiency and insulin resistance increase muscle protein degradation associated with the activation of the ubiquitin-proteasome system^51,52^. Our data showed that insulin decreased poly-ubiquitination of LRH-1 (Fig. 6C), suggesting the potential effects of insulin on suppression of ubiquitin-mediated degradation of LRH-1. This suggests one possible mechanism by which insulin may limit the action of DDB2 or other E3 ubiquitin ligase on targeting LRH-1 thus allowing the stabilization of LRH-1. As previously mentioned, CRY1 is also a target of the CUL4A-DDB1 complex for ubiquitination and degradation^46^. Similarly, Tong *et al*. have demonstrated that insulin increases CRY1 protein levels in liver cells, implying that the activity of CUL4A-DDB1 E3 ligase is inhibited by insulin^46^. Insulin can stimulate the transcription of sterol regulatory-element binding protein-1c (SREBP-1c), which transcriptionally regulates GCK expression, and thus SREBP-1c is considered as the major mediator of insulin action on GCK stimulation^53,54^. As LRH-1 is also a critical transcriptional activator of GCK and is involved in the hepatic glucose sensing system^6^, the upregulation of LRH-1 could be one of the important factors in mediating GCK expression response to the insulin signal. Furthermore, elevation of LRH-1 protein levels by insulin could augment GCK levels and allow rapid control of postprandial glucose and lipid metabolism.

DDB2 is likely to play a role in tumor suppression. Mice lacking DDB2 exhibited enhanced skin carcinogenesis and resistance to apoptosis in response to UV-irradiation^55^. DDB2 deficiency predisposes mice to the development of spontaneous malignant tumors^56^. Low expression of DDB2 is associated with skin cancer, ovarian cancer, and high-grade colon cancer^57–59^. In addition, overexpression of DDB2 reduces the migration and invasion of metastatic breast tumor cells by attenuating the activity of NF-κB^60^. In contrast, LRH-1 has been implicated in the development of some cancer cells including colon, pancreatic, gastric and, breast cancers^61–64^. LRH-1 can promote cell proliferation through interaction with the β-catenin to regulate the expression of cell-cycle regulators cyclin D1 and E1^65^. High expression of LRH-1 was detected in ER-positive and invasive ductal carcinoma of the breast^61,66^. LRH-1 has been shown to promote the motility and invasiveness of breast cancer cells^67^. Androgen receptor (AR) has been reported to be the target substrate of the DDB2-DDB1-CUL4A complex^34^. AR has an essential role in prostate cell proliferation and hence DDB2 can inhibit cell growth in AR-expressing prostate cancer cells. Similarly, the present study found that DDB2 promotes LRH-1 degradation and inhibits the transcriptional activity of LRH-1. The loss of DDB2 allows the rise of LRH-1 levels which may contribute to the development of some cancers such as colon and breast cancers. It will be of interest to determine the functional link between DDB2 and LRH-1 in cancer progression and pathogenesis.

In summary, we show that DDB2 interacts with LRH-1 to induce ubiquitination and degradation of LRH-1 via the CUL4-DDB1-DDB2 ligase complex, which is crucial for LRH-1 function in hepatic glucose metabolism. Moreover, lack of DDB2 results in dysregulation of glucose uptake, implicating that DDB2 could play a critical role in the regulation of glucose metabolism and offer a potential target in therapy of metabolic disorders.

## Methods

### Plasmids

The expression plasmids for mouse LRH-1, pFLAG-mLRH-1, and pEGFP-mLRH-1, were previously described^13^. Plasmid CDM8HIS-FLAG-hLRH-1 containing human LRH-1 cDNA was generously provided by Dr. David D. Moore (Department of Molecular and Cellular Biology, Baylor College of Medicine)^11^. The human LRH-1 cDNA was subcloned by PCR amplification from CDM8HIS-FLAG-hLRH-1 and inserted into vectors pEGFP-C2 and pcDNA3-FLAG to generate pEGFP-hLRH-1 and pFLAG-hLRH-1, respectively. The dominant negative Cullin plasmids (DN-CUL1, 2, 3, 4A, 4B, and 5) were gifts from Dr. J. Wade Harper (Addgene plasmids #15818 ~ #15823)^36^. The ubiquitin plasmids pcDNA3-HA-Ub for wild-type, K0, K48, K63, K48R, and K63R were kindly provided by Dr. C. Sasakawa (Department of Microbiology and Immunology, University of Tokyo)^68^. The double mutant pcDNA3-HA-UB-K48/K63R was generaged by site-directed mutagenesis PCR and verified by DNA sequencing. The expression plasmids for Myc-DDB2 and GST-DDB2 were generous gifts from Dr. Show-Li Chen (Graduate Institute of Microbiology, National Taiwan University College of Medicine, Taipei, Taiwan)^34^. The luciferase reporter Gck–Luc was generated by cloning upstream regions (−1076 to +144) of the mouse glucokinase gene into pGL3-Basic vector (Promega, WI, USA) and verified by DNA sequencing. The luciferase reporter SHP-Luc was generously provided by Dr. David J. Mangelsdorf (University of Texas Southwestern, Dallas, Texas)^9^.

### Cell culture, transfection, and treatment

HEK293T cells were maintained in Dulbecco’s modified Eagle’s medium (DMEM) supplemented with 10% fetal bovine serum. HepG2 cells were grown in MEM containing 10% fetal bovine serum and 1 mM sodium pyruvate. HepG2 cells that were stably transfected with pMyc-DDB2 or control vector pcDNA3-Myc were selected in 400 µg/ml G418 (Sigma-Aldrich, MO, USA) and then maintained in the presence of 200 µg/ml G418. Transfection was carried out using Turbofect (Thermo, MA, USA) for HEK293T cells and GenJet™ *In Vitro* DNA Transfection Reagent (Ver. II) (SignaGen, MD, USA) for HepG2 cells. For MG-132 (Calbiochem, MA, USA) treatment, after 24 h of transfection, HEK293T cells were incubated with 10 μM MG-132 for an additional 24 h. HepG2 cells were starved overnight with serum-free medium and then treated with MG-132 or insulin (Sigma-Aldrich) at specific concentrations and durations. In another experiment, cells were treated with 10 μM MLN4924 for 24 h (MLN4924 was provided by Dr. Kuo-How Huang, National Taiwan University, Taiwan).

### shRNA knockdown

The shRNA-expressing lentiviral plasmids (pLKO.1-shRNA) were obtained from National RNAi Core Facility (Academia Sinica, Taipei, Taiwan). CUL4A was efficiently targeted with construct TRCN0000320896, CUL4B was targeted with construct TRCN0000342588, DDB1 was targeted with construct TRCN0000303508, and DDB2 was targeted with construct TRCN0000083994 or TRCN0000083995. The shRNA construct (TRCN0000072223) targeting the LacZ was used as a control. Lentiviral particles were prepared as described previously^69^.

### Immunoblotting and immunoprecipitation

Cells were lysed in a buffer containing 20 mM Tris/HCl (pH 7.9), 137 mM NaCl, 10 mM NaF, 5 mM Ethylenediaminetetraacetic acid (EDTA), 1 mM ethylene glycol-bis(β-aminoethyl ether)-N,N,N′,N′-tetraacetic acid (EGTA), 1 mM Na_3_VO_4_, 10% (w/v) glycerol, 1% (v/v) Triton X-100, 1 mM sodium pyrophosphate, 0.1 mM β-glycerophosphate, 5 mM DTT (dithiothreitol), 2 mM phenylmethylsulfonyl fluoride (PMSF), and 10 μg/ml leupeptin, and were incubated on ice for 30 min. For poly-ubiquitin chain detection, the lysis buffer was supplemented with 20 mM N-Ethylmaleimide (Sigma-Aldrich). Sonication was followed by centrifugation at 13,000 × *g* for 30 min at 4 °C, the supernatant fraction was collected and either analyzed by western blotting or subjected to immunoprecipitation. Western blot analysis was performed by using anti-LRH-1-N^70^, anti-LRH-1 (sc-5995; Santa Cruz Biotechnology, TX, USA), anti-DDB1 (GeneTex, CA, USA), anti-DDB2 (R&D System, MN, USA), anti-CUL4A (GeneTex), anti-CUL4B (Proteintech, IL, USA), anti-Ub (Santa Cruz), anti-GFP (GeneTex), anti-GST (GeneTex), anti-FLAG (M2, Sigma-Aldrich), anti-HA (Sigma-Aldrich), anti-pAKT (Cell Signaling Technology, MA, USA), anti-GAPDH (Millipore, MA, USA), anti-Myc (Millipore), and anti-β-actin (Sigma-Aldrich) antibodies. For immunoprecipitation assays, the anti-LRH-1-N antibody was incubated with 30 μl of rProtein G agarose beads (Thermo) at 4 °C for 1 h, and the beads were collected by centrifugation 300 × *g* for 2 min, at 4 °C. Whole cell extracts were precleaned with 10 μl of rProtein G agarose beads at 4 °C for 2 h and then incubated overnight with antibody-bound beads at 4 °C, with gentle agitation. After washing with lysis buffer, beads were resuspended in protein sample buffer and analyzed for immunoblotting.

### GST pull-down assays

GST fusion proteins were expressed in *Escherichia coli* BL21 (DE3) cells by induction with 1 mM isopropyl-β-D-thiogalactopyranoside (IPTG) for 4 h, at 30 °C. Cells were pelleted, and then resuspended in extraction buffer (2 mM EDTA, 2 mM EGTA, 2 mM DTT, 200 μg/ml lysozyme, 1 mM PMSF, 10 μg/ml aprotinin, and 10 μg/ml leupeptin) on ice for 30 minutes. After sonication and centrifugation, the GST fusion proteins in the supernatant were incubated overnight with glutathione-sepharose beads (GE healthcare Life Sciences, PA, USA) at 4 °C. After three washes with Phosphate buffered saline (PBS), bead-bound GST- tagged proteins were incubated overnight with protein lysates at 4 °C. The bound proteins were washed with PBS/Triton X-100 and then subjected to immunoblotting.

### Cycloheximide chase experiment

HEK293T cells were cotransfected with pFLAG-hLRH-1 and pMyc-DDB2 or control vector pcDNA3-Myc. 24 h after transfection, cells were treated with 100 μg/ml cycloheximide (Sigma-Aldrich). Cell lysates were collected at the specified time points and analyzed by immunoblotting.

### Luciferase assay

HEK293T cells were subcultured 24 h before transfection onto 24-well plates at a density of 10^5^ cells/well. Cells were transfected with 100 ng of pFLAG-mLRH-1, 100 ng of pMyc-DDB2, 100 ng of reporter pGck-Luc or pSHP-Luc, and 2 ng of control reporter phRLuc, using Turbofect (Thermo). After 24 h, the cells were harvested and luciferase activities were determined using the Dual-Glo Luciferase Assay System (Promega). The results were normalized to internal Renilla luciferase activities. The significance of differences between group means was determined using the Student’s *t*-test.

### RNA extraction and quantitative reverse transcription PCR (RT-qPCR)

Total RNA was extracted using TRIzol reagent (Thermo), and then reverse transcribed into cDNA by First Strand cDNA Synthesis Kit (Thermo). Quantitative PCR reactions were performed using SYBR® Green PCR Master Mix (Thermo), in an ABI 7500 or StepOnePlus^TM^ machine (Thermo). The primer used were as follows: *LRH-1* forward 5′-AGAAGGTGTCCAGGAACAAGTCA-3′, and reverse 5′-CTCTGTCTGCTGCGGGTAGTTAC-3′; *CYP7A1* forward 5′-ACACCATTCCAGCGACTTTCTG-3′, and reverse 5′-AGGCACTGGAAAGCCTCAGC-3′; *SHP* forward 5′-CAAGAAGATTCTGCTGGAGG-3′, and reverse: 5′-GATGTCAACATCTCCAATG-3′; *GCK* forward 5′-GGTGGCAATGGTGAATGACA-3′, and reverse 5′-CTCGCACTGATGGTCTTCGTAGT-3′; *GAPDH* forward 5′-AATCCCATCACCATCTTCCA-3′, and reverse 5′-TGGACTCCACGACGTACTCA-3′; *ACTB* forward 5′-GGGAAATCGTGCGTGAC-3′, and reverse 5′-CAAGAAGGAAGGCTGGAA-3′.

### Glucose assay

Cells were cultured in fresh MEM medium without sodium pyruvate for 24 h. Cell culture medium was collected and cells were trypsinized and counted. Glucose concentration in the medium was measured by Glucose Colorimetric Assay Kit II (Biovision, CA, USA). Glucose consumption was determined by a decrease in the amount of glucose in culture medium after incubation. Glucose consumption and lactate production were normalized to cell numbers. The experiments were performed with 4 replicates and repeated 3 times.

### Glucose-6-phosphate assay

Cells were cultured in fresh MEM medium for 2 h before harvest. Cells were collected and homogenized with a Dounce grinder on ice. The samples were passed through Microcon 10K centrifugal filter units (Millipore) to separate the proteins. Glucose-6-phosphate levels were determined with the Glucose-6-phosphate Colorimetric Assay Kit (Biovision). The experiments were performed with 2 replicates and were repeated five times.

### Statistical analysis

The statistical analysis was performed by the Student’s *t* test using GraphPad Prism 6 software (GraphPad Software). Values with *P* < 0.05 were considered statistically significant.

## Supplementary information

Supplementary information
