# Supplementary material for: Regulation of liver receptor homologue-1 by DDB2 E3 ligase activity is critical for hepatic glucose metabolism
Source: Sci Rep. 2019 Mar 28;9:5304. doi: 10.1038/s41598-019-41411-x (PMC6438966; doi:10.1038/s41598-019-41411-x)
Supplement: Supplementary file 1 — Supplementary information [file 41598_2019_41411_MOESM1_ESM.pdf]

Supplementary Information

Regulation of liver receptor homologue-1 by DDB2 E3 ligase activity is critical for hepatic glucose metabolism

Tsai-Chun Lai and Meng-Chun Hu

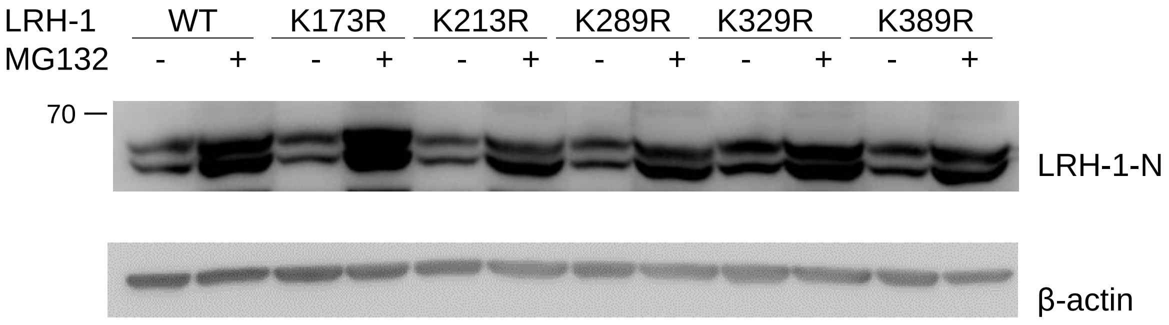

Supplementary Figure S1. Effect of lysine mutations on mLRH-1 stability.

(A) Wild-type or lysine-to-arginine mutations of FLAG-mLRH-1 were transfected into HEK293T cells. After 24 h, cells were treated with DMSO (vehicle control) or MG-132 (10 μM) for 24 h. Cell lysates were analyzed by immunoblotting with anti-LRH-1-N antibody. β-actin was used as the loading control.

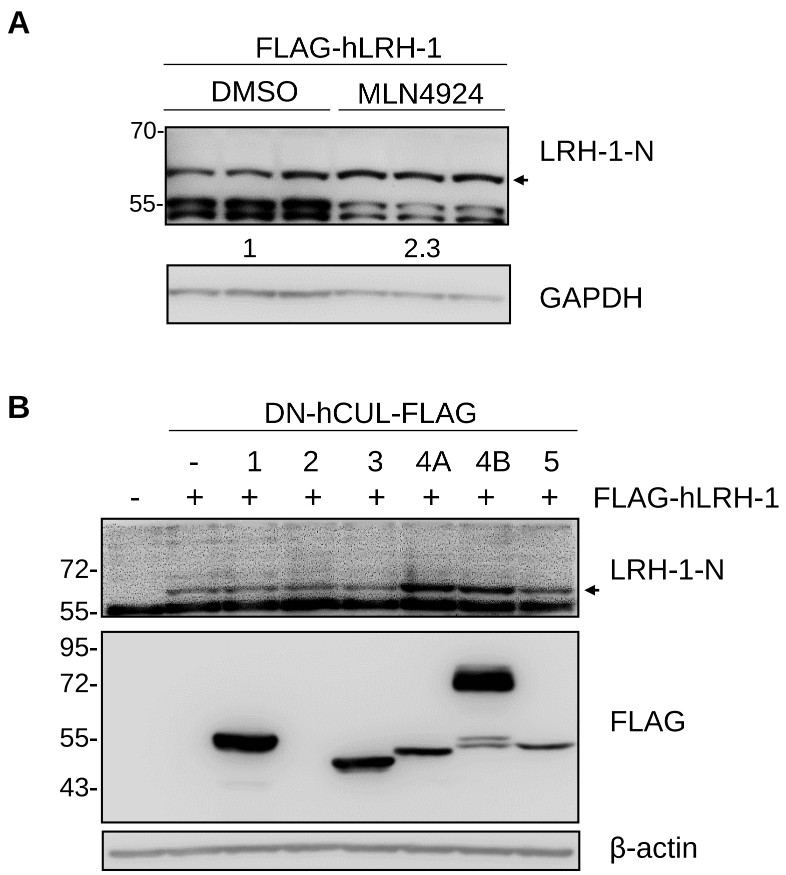

Supplementary Figure S2. CRL4 ubiquitin ligases regulate hLRH-1 protein degradation.

(A) FLAG-hLRH-1 was transfected into HEK293T cells. After 24 h, cells were treated with DMSO (vehicle control) or MLN4924 (10 μM) for 24 h. Cell lysates were analyzed by immunoblotting with anti-LRH-1-N. Numbers below the blot are the average of densitometric values normalized to those of GAPDH. (B) FLAG-hLRH-1 was co-transfected with FLAG-tagged dominant negative constructs of Cullin 1, 2, 3, 4A, 4B, and 5 or empty vector (-) into HEK293T cells. After 48 h, cell lysates were subjected to immunoblotting analysis with anti-LRH-1-N or anti-FLAG antibodies. β-actin was used as the loading control.

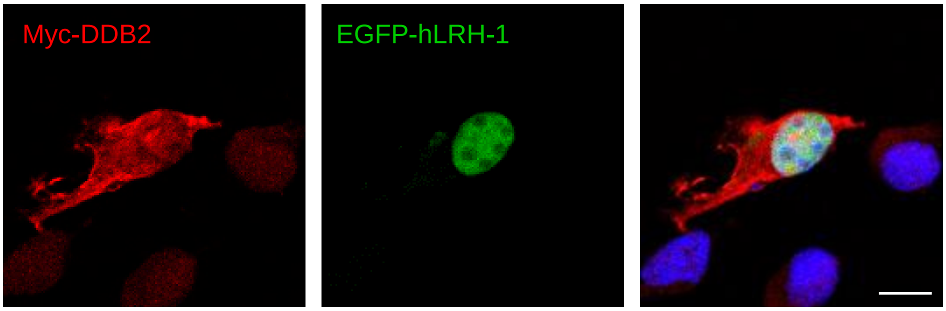

Supplementary Figure S3. The co-localization of LRH-1 with DDB2.

EGFP-hLRH-1 and Myc-DDB2 were co-transfected into HEK293T cells and immunostained with the anti-Myc antibody. Images were obtained by confocal microscopy. Scale bar = 10 μm.

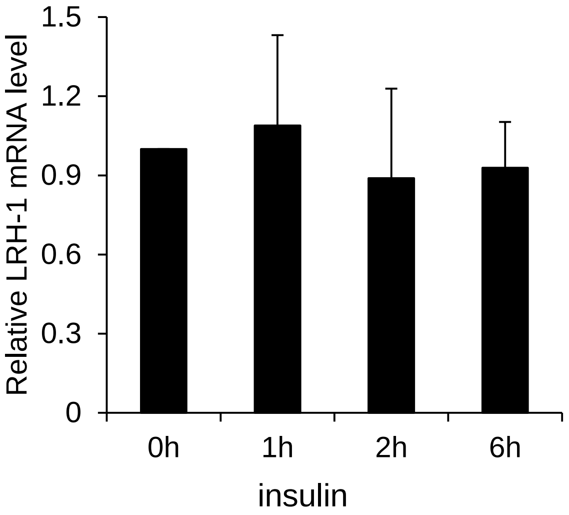

Supplementary Figure S4. No effect of insulin on LRH-1 mRNA levels.

HepG2 cells were serum-starved overnight and incubated with insulin (75 ng/ml) or vehicle control for indicated periods of time. mRNA levels of LRH-1 were quantified by RT-qPCR. Data were expressed as mean ± SD relative to vehicle control (0 h) of three independent experiments.

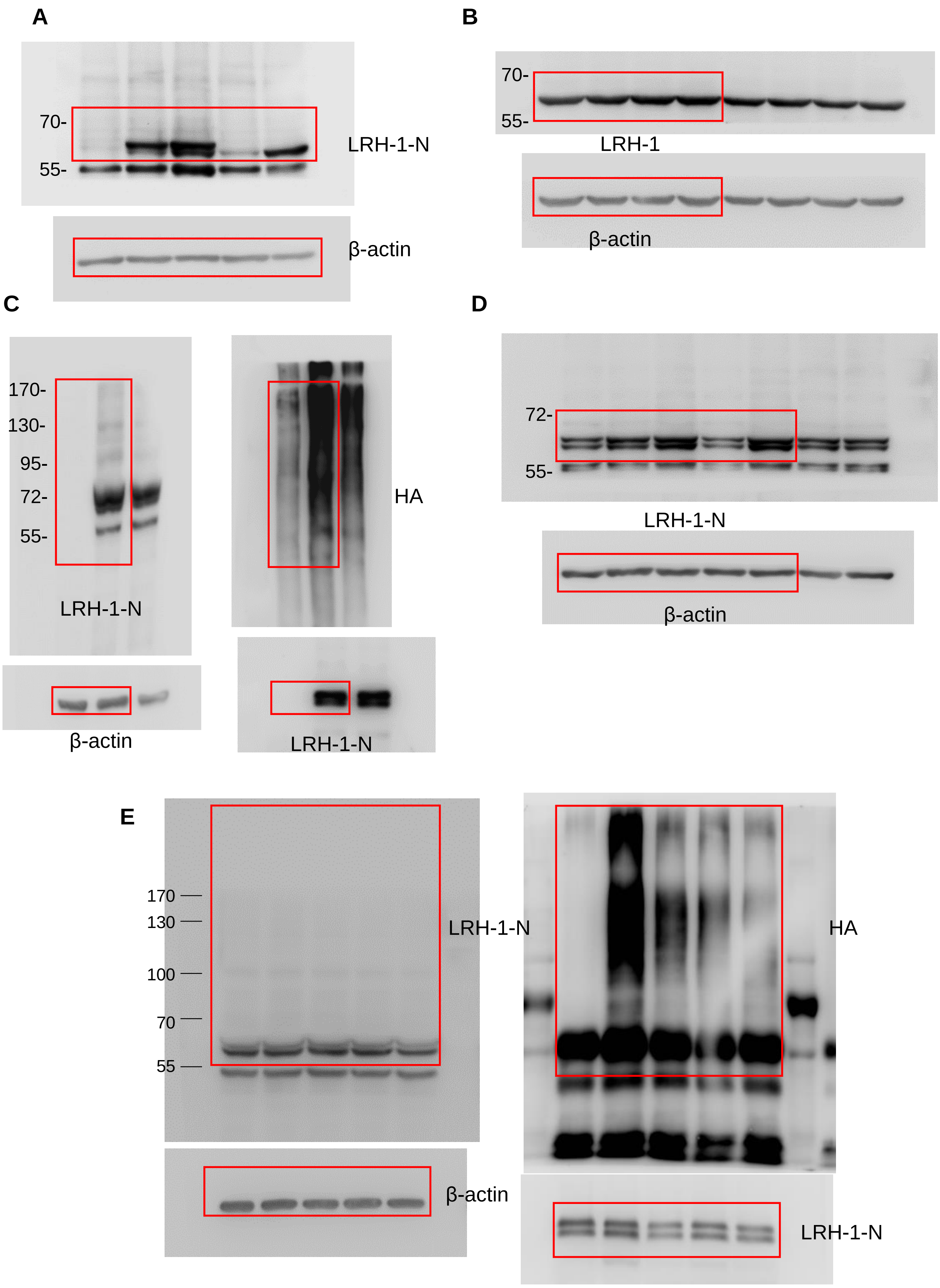

Supplementary Figure S5. Uncropped images of blots presented in the main Figure 1.

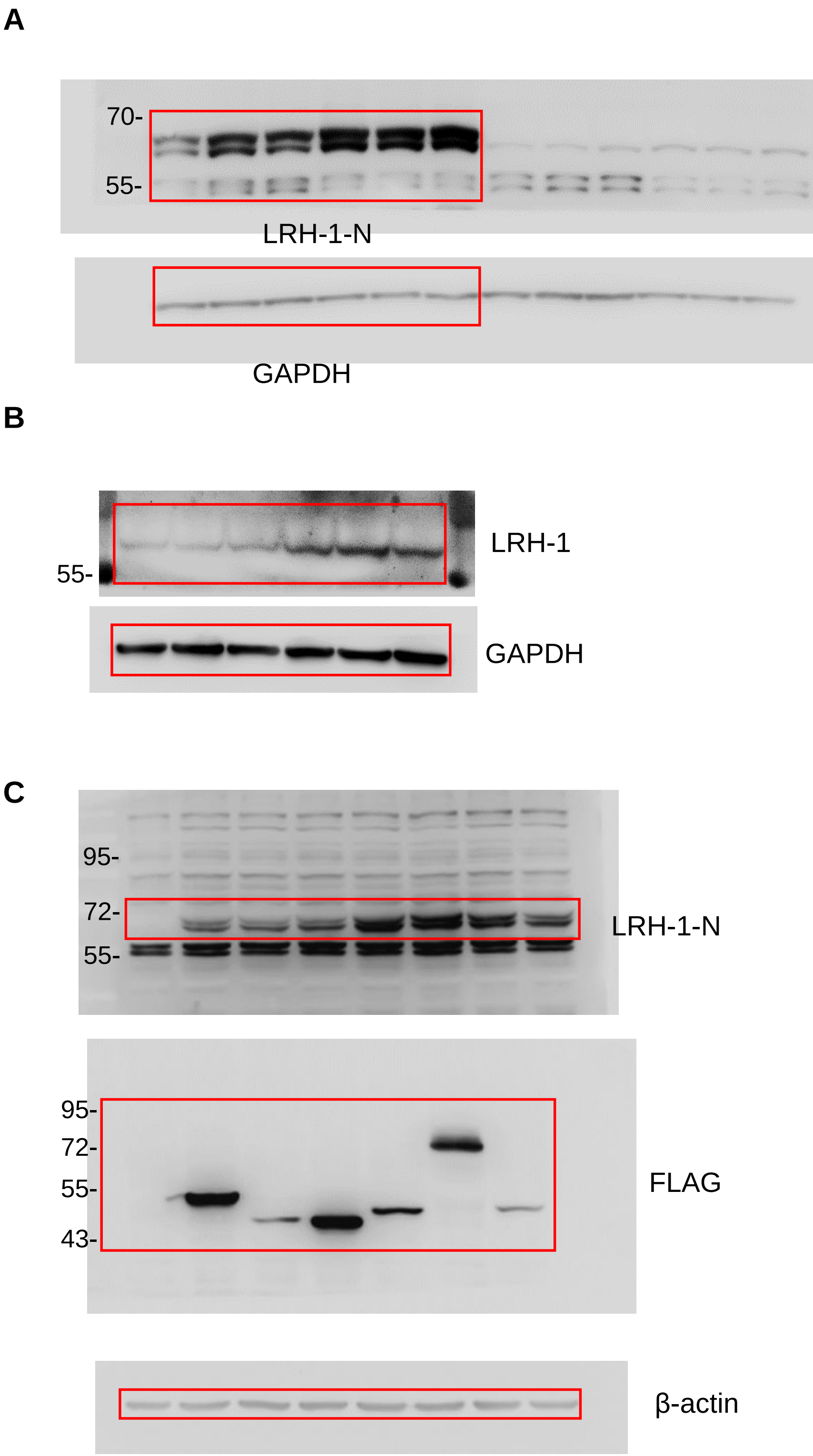

Supplementary Figure S6. Uncropped images of blots presented in the main Figure 2.

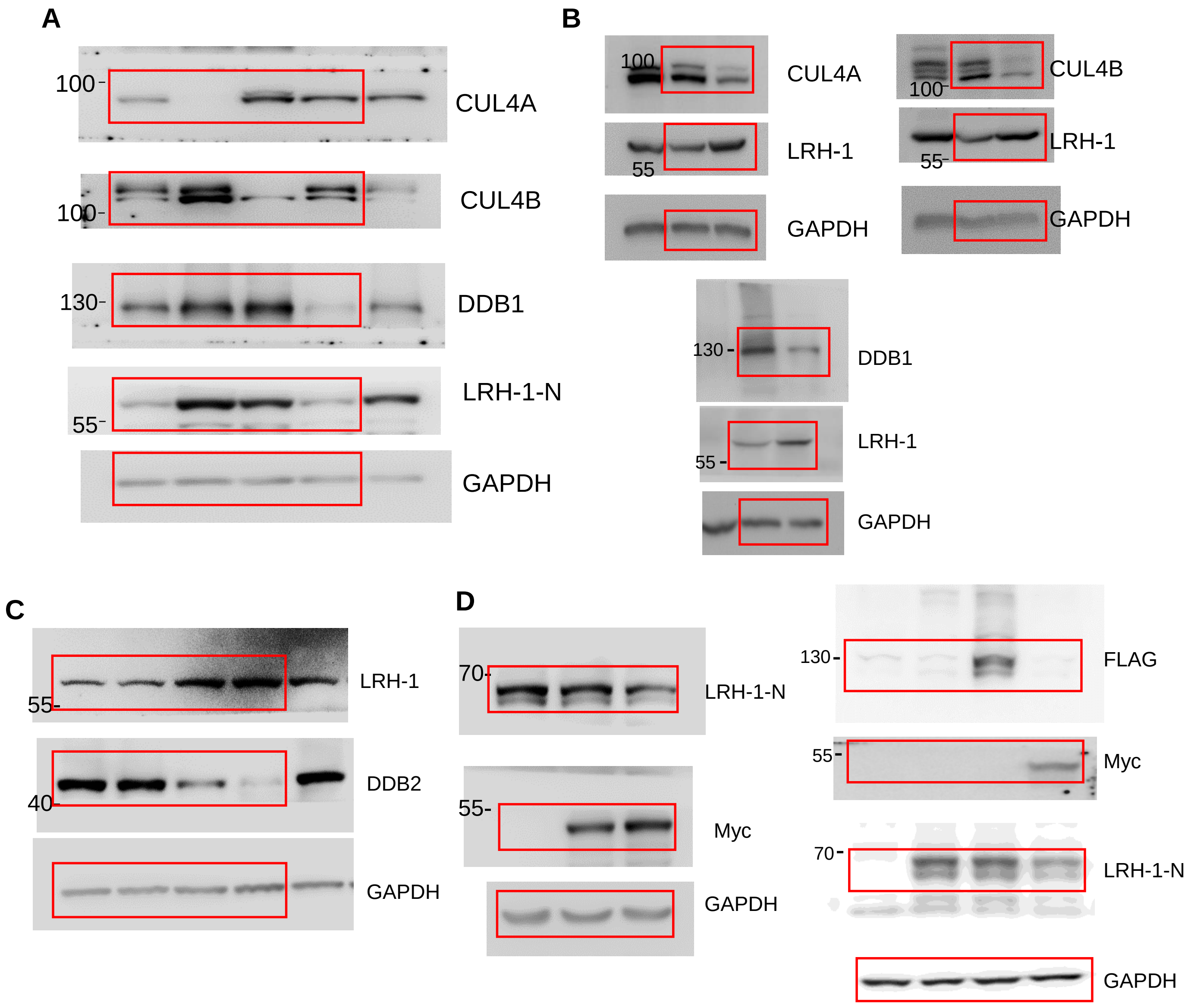

Supplementary Figure S7. Uncropped images of blots presented in the main Figure 3.

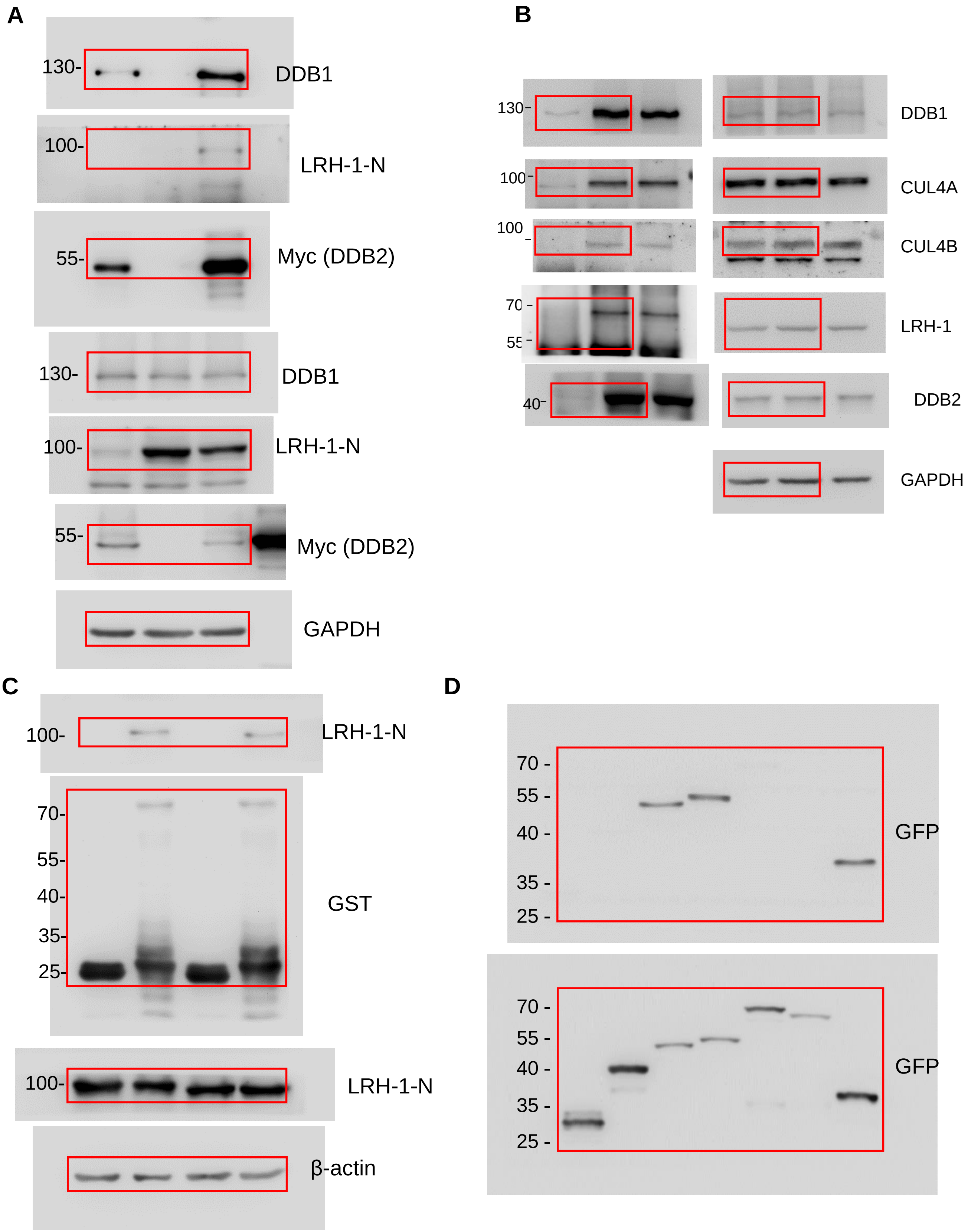

Supplementary Figure S8. Uncropped images of blots presented in the main Figure 4.

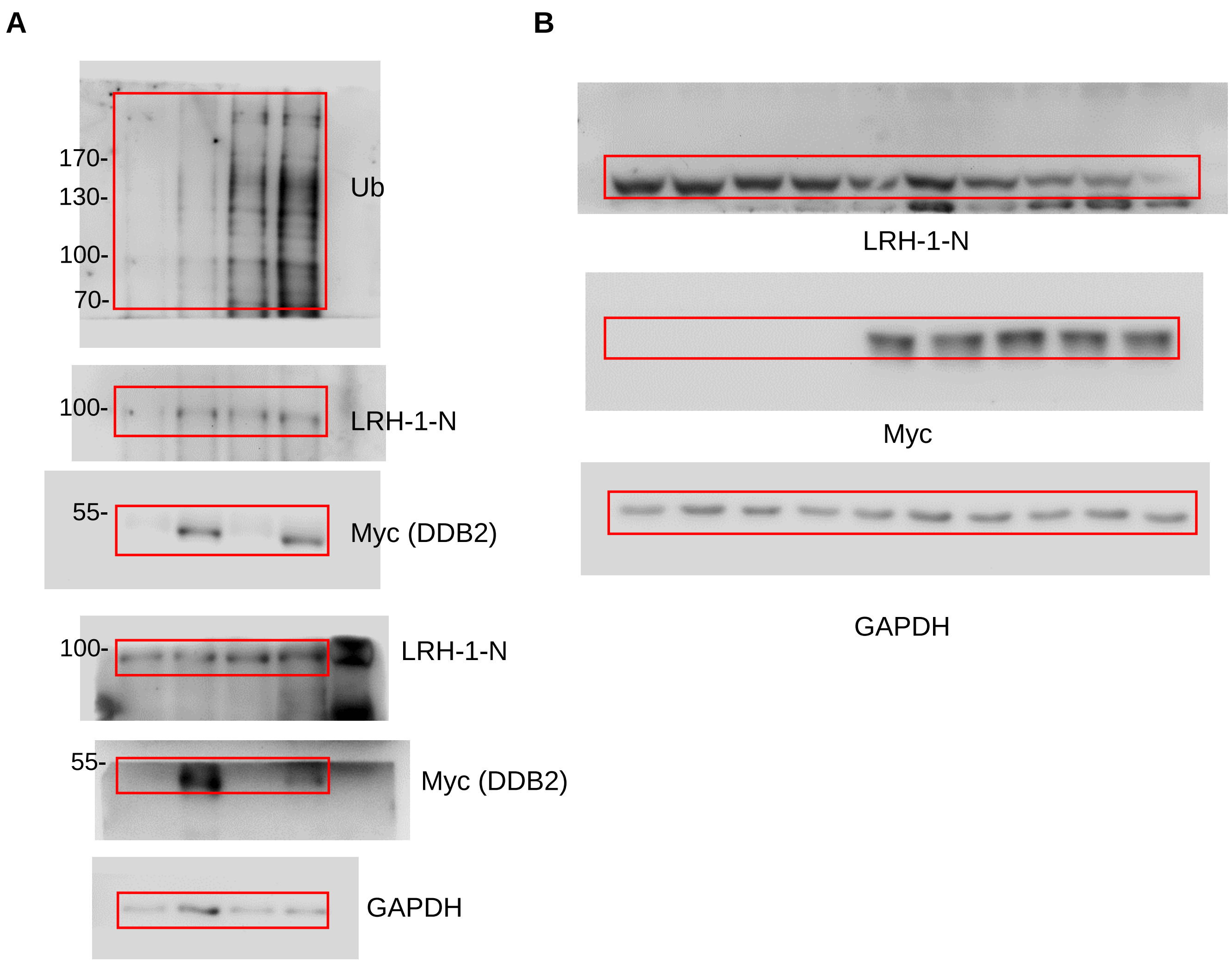

Supplementary Figure S9. Uncropped images of blots presented in the main Figure 5.

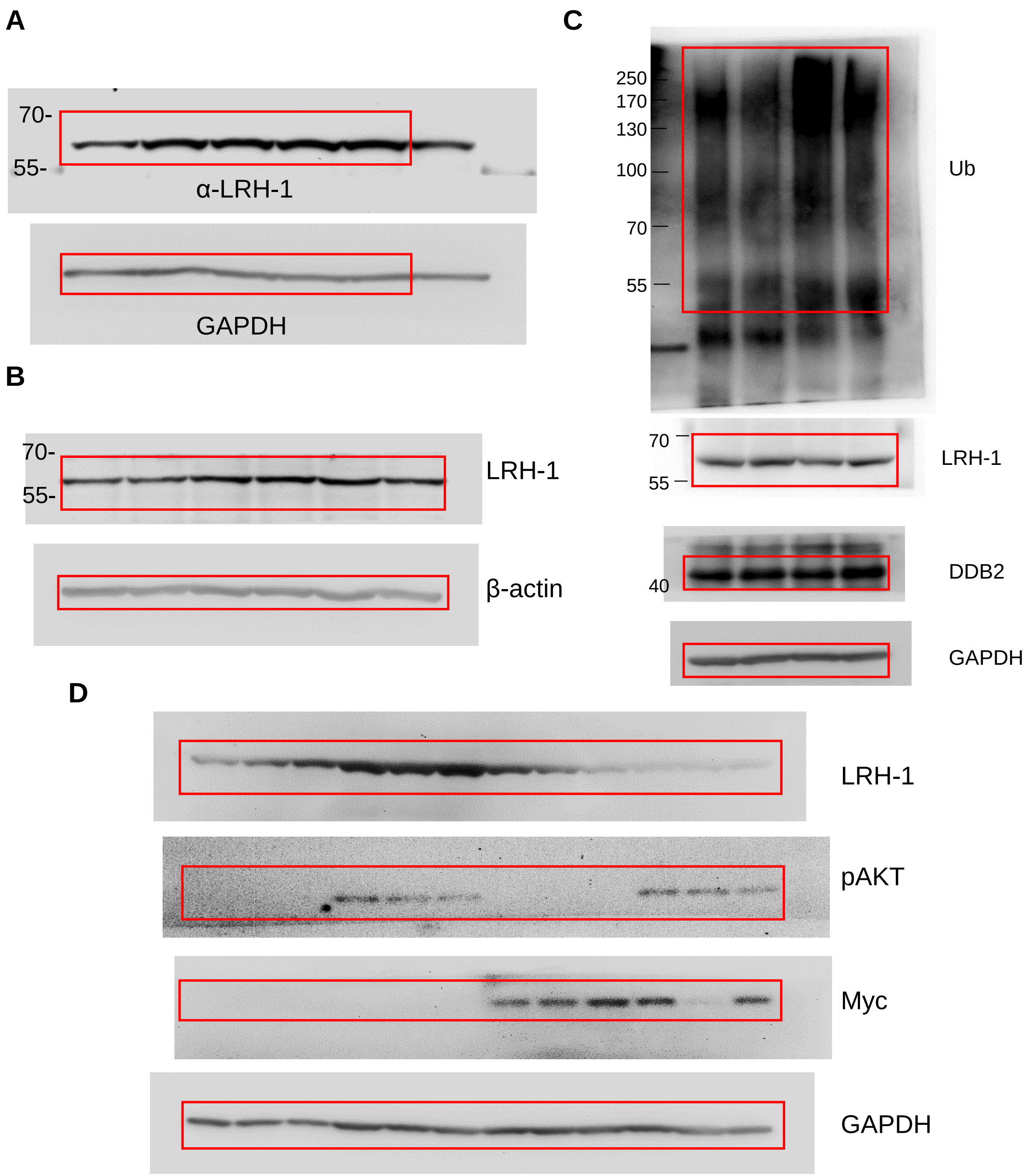

Supplementary Figure S10. Uncropped images of blots presented in the main Figure 6.

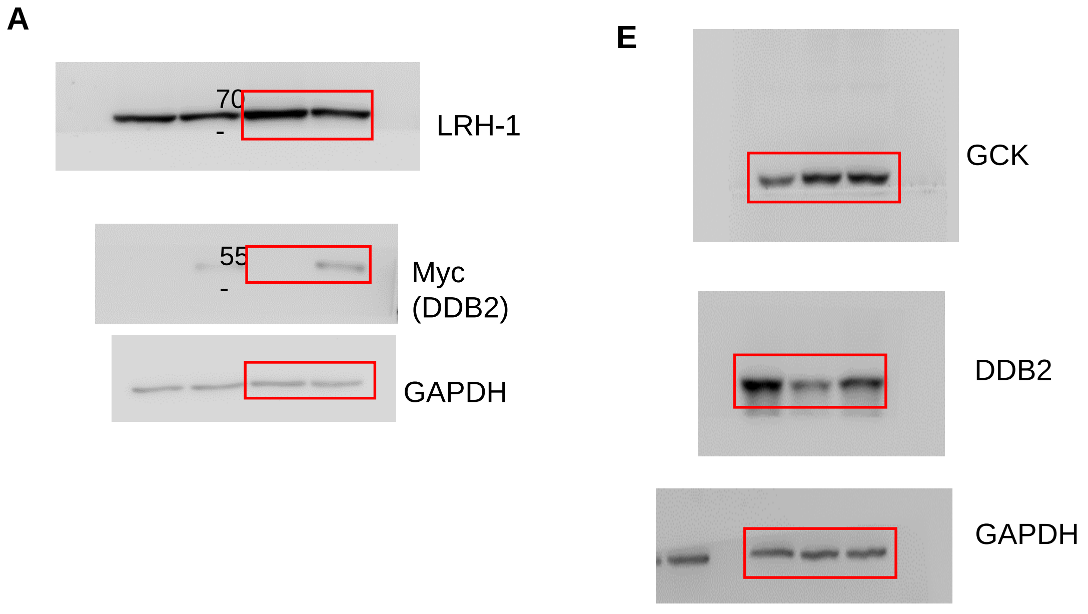

Supplementary Figure S11. Uncropped images of blots presented in the main Figure 7.
